# Hydrogenation of Inorganic Metal Carbonates: A Review on Its Potential for Carbon Dioxide Utilization and Emission Reduction

**DOI:** 10.1002/cssc.201801356

**Published:** 2018-08-29

**Authors:** Susanne Lux, Georg Baldauf‐Sommerbauer, Matthäus Siebenhofer

**Affiliations:** ^1^ Institute of Chemical Engineering and Environmental Technology Graz University of Technology Inffeldgasse 25C/II 8010 Graz Austria

**Keywords:** decarboxylation, hydrogenation, heterogeneous catalysis, surface chemistry, transition metals

## Abstract

Carbonaceous minerals represent a valuable and abundant resource. Their exploitation is based on decarboxylation at elevated temperature and under oxidizing conditions, which inevitably release carbon dioxide into the atmosphere. Hydrogenation of inorganic metal carbonates opens up a new pathway for processing several metal carbonates. Preliminary experimental studies revealed significant advantages over conventional isolation technologies. Under a reducing hydrogen atmosphere, the temperature of decarboxylation is significantly lower. Carbon dioxide is not directly released into the atmosphere, but may be reduced to carbon monoxide, methane, and higher hydrocarbons, which adds value to the overall process. Apart from metal oxides in different oxidation states, metals in their elemental form may also be obtained if transition‐metal carbonates are processed under a hydrogen atmosphere. This review summarizes the most important findings and fields of the application of metal carbonate hydrogenation to elucidate the need for a detailed investigation into optimized process conditions for large‐scale applications.

## 1 Introduction

Carbon dioxide (CO_2_) is an abundant chemical species. On earth, it is present in all three aggregate states: in gaseous form in the atmosphere (approximately 2.5×10^12^ tons), in the dissolved state in the hydrosphere (approximately 10^16^ tons), and in the solid state fixed in carbonate rocks (approximately 10^14^ tons).^1^ Hence, more (by a factor of 10^4^) terrestrial carbon is fixed in carbonate rocks in the earth's crust than is actually present in the gaseous atmosphere. Carbonate rocks have been known as a valuable and abundant resource for a long time. The history of inorganic metal carbonate chemistry dates back to the early ages of solid‐state chemistry. The thermal decomposition, meaning decarboxylation reactions of metal carbonates in an oxidizing atmosphere, also known as calcination, roasting, or burning, depending on the metal species involved, represent processes that were developed first on experience, later on research, and finally on technological optimization in industrial production plants. Cato, for instance, mentioned the burning of limestone (calcite, CaCO_3_) in kilns in 184 B.C. In the 1700s, Joseph Black gave the first systematic technical explanation of the calcination of limestone, also including the evolution of gaseous carbon dioxide.^2^ Lime (CaO) is one of the cheapest and most widely used alkalizing chemicals and the main constituent of cement production. The production of magnesium oxide (magnesia, MgO) through calcination of magnesite (MgCO_3_) for refractory manufacture was established over 100 years ago in Austria.^3^ The roasting of iron carbonate (siderite, FeCO_3_) is used for iron and steel production in areas with vast mineral iron carbonate reserves, such as Austria^4^ and China.^5^, ^6^ The cement industry, iron production, and manufacture of magnesia sinter make use of large quantities of the respective metal carbonates. Further industrial applications of metal carbonates of, for instance, nickel, cobalt, manganese, and zinc, or more precisely of their respective oxides or the metals in their elemental form, include the production of catalysts (catalytic material and porous support), pigments and glass, the ceramics industries, and the electronics industries.^3^, ^7^, ^8^

During the decarboxylation of metal carbonates to yield the respective metal oxide (MeO), inevitably one mole of CO_2_ and/or carbon monoxide (CO) evolve per mole of metal oxide formed. The metal oxide, in turn, gives access to retransformation into the corresponding carbonate through CO_2_ uptake. This characteristic provides the basis for carbon capture and storage (CCS) technologies, referred to as mineral carbonation, in which CO_2_ is fixed to and stored as carbonate minerals, mainly in the form of calcium and magnesium carbonate.^9^

Whereas the decarboxylation products of main‐group elements (e.g., alkaline‐earth‐metal carbonates) are the corresponding metal oxides and CO_2_ [Eq. (1)], the decomposition of transition‐metal carbonates follows a more complex reaction pathway [Eq. (2)] because redox processes may take place. The redox behavior of the transitions metals allows the reduction of CO_2_ to CO by means of thermodynamic fundamentals. The solid products can be metal oxides and mixtures of metal oxides that adopt different oxidation states.^10^

(1) $$\mathrm{MeCO}{}_{3}↔\mathrm{MeO}+\mathrm{CO}{}_{2}$$

(2) $$\mathrm{MeCO}{}_{3}↔\mathrm{MeO}{}_{1+x}+\left(1-x\right)\;\mathrm{CO}{}_{2}+x\;\mathrm{CO}$$

In metal carbonate decarboxylation, the reaction conditions, especially the nature of the gas atmosphere, play a crucial role in the course of the reaction. If carried out in a reducing atmosphere with hydrogen (H_2_), a fascinating reaction network is observed. Equation (3) shows the reaction for the hydrogenation of alkaline‐earth‐metal carbonates, whereas Equation (4) gives a potential reaction pathway for the hydrogenation of transition‐metal carbonates. Apart from metal oxides and mixtures of metal oxides in different oxidation states, metals in their elemental form can be formed as solid products from transition‐metal carbonates. Gaseous products may include methane (CH_4_) in addition to or in place of CO_2_ and CO.^10^ Some citations even report the formation of higher hydrocarbons (C_1_–C_*x*_).^11^, ^12^, ^13^ In this context, it is noteworthy that an admixture of iron oxides enables the direct conversion of calcium carbonate into C_1_–C_3_ hydrocarbons.^13^

(3) $$\mathrm{MeCO}{}_{3}+4\;H{}_{2}↔\mathrm{MeO}+\mathrm{CH}{}_{4}+2\;H{}_{2}O$$

(4) $$\mathrm{MeCO}{}_{3}+\left(1+y+4\;z\right)\;H{}_{2}↔\mathrm{MeO}{}_{1-x}+\left(1-y-z\right)\;\mathrm{CO}{}_{2}\;\;\;\;\;\;\;+y\;\mathrm{CO}+z\;\mathrm{CH}{}_{4}+\left(x+y+2\;z\right)\;H{}_{2}O$$

Transition metals are well established catalysts in Fischer–Tropsch (FT) synthesis,^14^, ^15^, ^16^ and the water‐gas shift reaction.^17^, ^18^, ^19^, ^20^ FT synthesis, namely, the catalytic polymerization and hydrogenation of CO, gives access to the synthesis of hydrocarbons (alkanes, alkenes, and oxygenated hydrocarbons) from syngas, CO and H_2_.^21^ The reaction for the synthesis of alkanes is shown in Equation (5) as an example. The reverse water‐gas shift reaction yields CO from CO_2_ through reduction with hydrogen [Eq. (6)]. Consequently, the presence of transition metals in metal carbonate hydrogenation opens up a pathway for the synthesis of hydrocarbons, instead of simply releasing CO_2_ into the gas phase.

(5) $$n\;\mathrm{CO}+\left(2\;n+1\right)\;H{}_{2}↔C{}_{n}H{}_{2\;n+2}+n\;H{}_{2}O$$

(6) $$\mathrm{CO}{}_{2}+H{}_{2}↔\mathrm{CO}+H{}_{2}O$$

In addition to different gaseous products that are released from metal carbonates under reducing conditions, changing the gaseous atmosphere from inert to reducing also has an effect on the morphology of the solid products.^22^ In most cases, the reducing agent in reductive metal carbonate decarboxylation is hydrogen. Until now, the production of hydrogen on an industrial scale has mainly (>95 %) been based on fossil fuels, for instance, through steam reforming of methane or gasification of coal and hydrocarbons, which, apart from hydrogen, generates CO_2_.^23^, ^24^ Apart from conventional production routes, hydrogen can also be produced renewably and sustainably from various sources (e.g., water electrolysis or water splitting through photocatalysis,^25^, ^26^ solar thermal production,^27^ photosynthesis by algae,^28^ reforming of biomass^29^). Hydrogen storage is challenging, however, and requires a completely new distribution system.^23^

Whereas the decomposition of various metal carbonates in a vacuum or under an oxidizing or inert atmosphere has been well investigated and reported (e.g., for FeCO_3_ in vacuum,^30^ oxygen,^31^, ^32^, ^33^ and nitrogen^34^, ^35^, ^36^), information on metal carbonate hydrogenation is scarce. Reduction of carbonates in aqueous solution is also well described in the literature and is not covered herein.^37^, ^38^, ^39^, ^40^, ^41^, ^42^ Metal carbonate hydrogenation is not only challenging from a reaction mechanism point of view, but also features some characteristics that render it promising in terms of carbon capture and utilization (CCU), hydrogen storage, and novel production technologies in metallurgy. The purpose herein is to provide a comprehensive literature review on metal carbonate hydrogenation and to discuss potential applications. After describing their natural occurrence (Section 2), thermodynamics of metal carbonate hydrogenation are specified (Section 3). Experimental studies that have been conducted so far are listed in Section 4. After summing up the main benefits of metal carbonate hydrogenation (Section 5), feasible process options are illustrated and evaluated (Section 6). Finally, Section 7 identifies future needs for detailed research.

## 2 Natural Occurrence

Inorganic carbonates, characterized by planar [CO_3_]^2−^ complexes with metal ions, form one of the most important mineral groups. Taxonomy is based on cations and/or anions outside of the [CO_3_]^2−^ complexes. Water‐free mineral carbonates may feature calcite‐ (trigonal, scalenohedric), dolomite‐ (trigonal, rhombohedral), and aragonite‐type (orthorhombic) structures. Carbonates with additional anions may contain OH^−^ anions or water.

### 2.1 Main‐group elements

#### 2.1.1 Alkaline metal carbonates

Mineral lithium carbonate (Li_2_CO_3_, zabuyelite) occurs in the lithosphere as an ore companion, mainly imbedded in halite in rock salt and in saline lakes. Sodium carbonate (Na_2_CO_3_, natrite, also known as soda) appears in saline lakes and minerals such as trona Na_3_(CO_3_)(HCO_3_)⋅2 H_2_O and natron Na_2_CO_3_⋅10 H_2_O. It undergoes a rapid change superficially in air to thermonatrite (Na_2_CO⋅H_2_O). Potassium carbonate (K_2_CO_3_) is called potash because it was historically produced by treating wood ash with water in a pot. There are no K_2_CO_3_‐containing ores known that would be worth mining. It is generally produced from electrolytically produced KOH and CO_2_. Rubidium (Rb_2_CO_3_) and cesium (Cs_2_CO_3_) carbonate are naturally found together in low concentrations accompanying sodium‐ and potassium‐containing ores. Similar to the other alkaline metals, they also collect in saline lakes. Francium isotopes (Fr_2_CO_3_) are radioactive and only found in traces in the lithosphere.^43^, ^44^

#### 2.1.2 Alkaline‐earth‐metal carbonates

Beryllium carbonate (BeCO_3_) barely exists in minerals because it is easily decomposed into beryllium oxide and CO_2_, and therefore, only stable in a CO_2_ atmosphere. It exists naturally in the mineral niveolinite NaBe(CO_3_)_2_(OH)⋅2 H_2_O. Magnesium carbonate (MgCO_3_) is found in the lithosphere as magnesite MgCO_3_ and dolomite CaMg(CO_3_)_2_, such as in the Southern Alps—the Dolomites. Mining of magnesite is one of the main resources for magnesium carbonate. Calcium carbonate (CaCO_3_) mainly forms calcite CaCO_3_ and dolomite CaMg(CO_3_)_2_. Mining of these minerals is the main source for calcium carbonate. Three different naturally occurring crystal structures of calcium carbonate exist: calcite, aragonite, and vaterite. In calcite, the central Ca^2+^ ion is coordinated by six oxygen atoms, whereas in aragonite nine oxygen atoms coordinate the central Ca^2+^ ion. Strontium carbonate (SrCO_3_) is found in the naturally occurring mineral strontianite. Barium carbonate (BaCO_3_) forms in the lithosphere as witherite.^43^, ^44^

#### 2.1.3 Further main‐group metals

Lead carbonate (PbCO_3_) is found as cerussite. Caustic aluminum carbonate is found in the mineral dawnsonite NaAl(CO_3_)(OH)_2_.^43^, ^44^

### 2.2 Transition metals

Manganese is present in the lithosphere nearly as frequently as carbon or phosphorus. Manganese carbonate (MnCO_3_) is found in the mineral rhodochrosite and as an ore companion of iron. Iron carbonate (FeCO_3_) is found in the lithosphere as siderite and ankerite (Ca(Mg,Fe)[CO_3_]_2_), for example, at the Erzberg in Styria, Austria, and in China. Cobalt occurs in diverse forms in the lithosphere, often as an ore companion. Nevertheless, no major cobalt carbonate (CoCO_3_)‐containing ore is known. Cobalt carbonate can be produced by precipitation of water‐soluble cobalt(II)salts with alkaline‐earth carbonates. Nickel exists in diverse forms in the lithosphere, but no nickel carbonate ores are known. The industrially most important nickel carbonate is caustic nickel carbonate, 2 NiCO_3_ **⋅**3 Ni(OH)_2_ **⋅**4 H_2_O, produced by precipitation of aqueous nickel sulfate with sodium carbonate. Caustic nickel carbonate can be dehydrated to give anhydrous nickel carbonate or the hexahydrate NiCO_3_ **⋅**6 H_2_O. Copper carbonate (CuCO_3_) occurs naturally as azurite 2 CuCO_3_ **⋅**Cu(OH)_2_ (blue) and malachite CuCO_3_⋅Cu(OH)_2_ (green). Silver carbonate (Ag_2_(CO_3_)_3_) is not found in minerals, but precipitates from water by using soluble silver species and alkaline‐metal carbonates, such as soda. Zinc carbonate (ZnCO_3_) exists as smithsonite. Cadmium carbonate (CdCO_3_) is mostly found as an ore companion of smithsonite.^43^, ^44^

## 3 Thermodynamics of Metal Carbonate Hydrogenation

**Figure 1:**
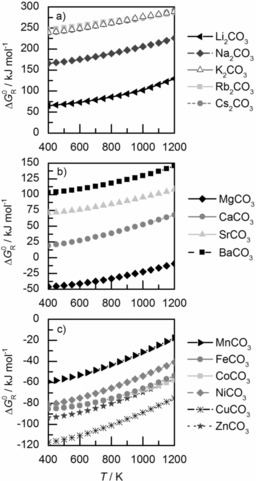
Standard free energies of reaction, Δ*G*_R_^0^, for methane formation through hydrogenation of metal carbonates between 400 and 1200 K at ambient pressure calculated with HSC Chemistry 8 software; a) alkaline‐metal carbonates: Me_2_CO_3_+4 H_2_↔Me_2_O+CH_4_+2 H_2_O, b) alkaline‐earth‐metal carbonates, and c) transition‐metal carbonates: MeCO_3_+4 H_2_↔MeO+CH_4_+2 H_2_O.

Thermodynamic analysis of alkaline, alkaline‐earth, and transition‐metal carbonates between 400 and 1200 K at ambient pressure shows increasing standard free energies of reaction, Δ*G*_R_^0^, for methane formation with increasing temperature. Due to strongly positive Δ*G*_R_^0^ values (>60 kJ mol^−1^), methane formation is not possible through hydrogenation of alkaline‐metal carbonates (Figure 1 a). Among alkaline‐earth‐metal carbonates, only hydrogenation of MgCO_3_ features a negative Δ*G*_R_^0^, favoring CH_4_ formation (Figure 1 b). Hydrogenation of the transition‐metal carbonates MnCO_3_, FeCO_3_, CoCO_3_, NiCO_3_, CuCO_3_, and ZnCO_3_ to their respective bivalent oxides, CH_4_, and H_2_O is favorable (Figure 1 c).

**Figure 2:**
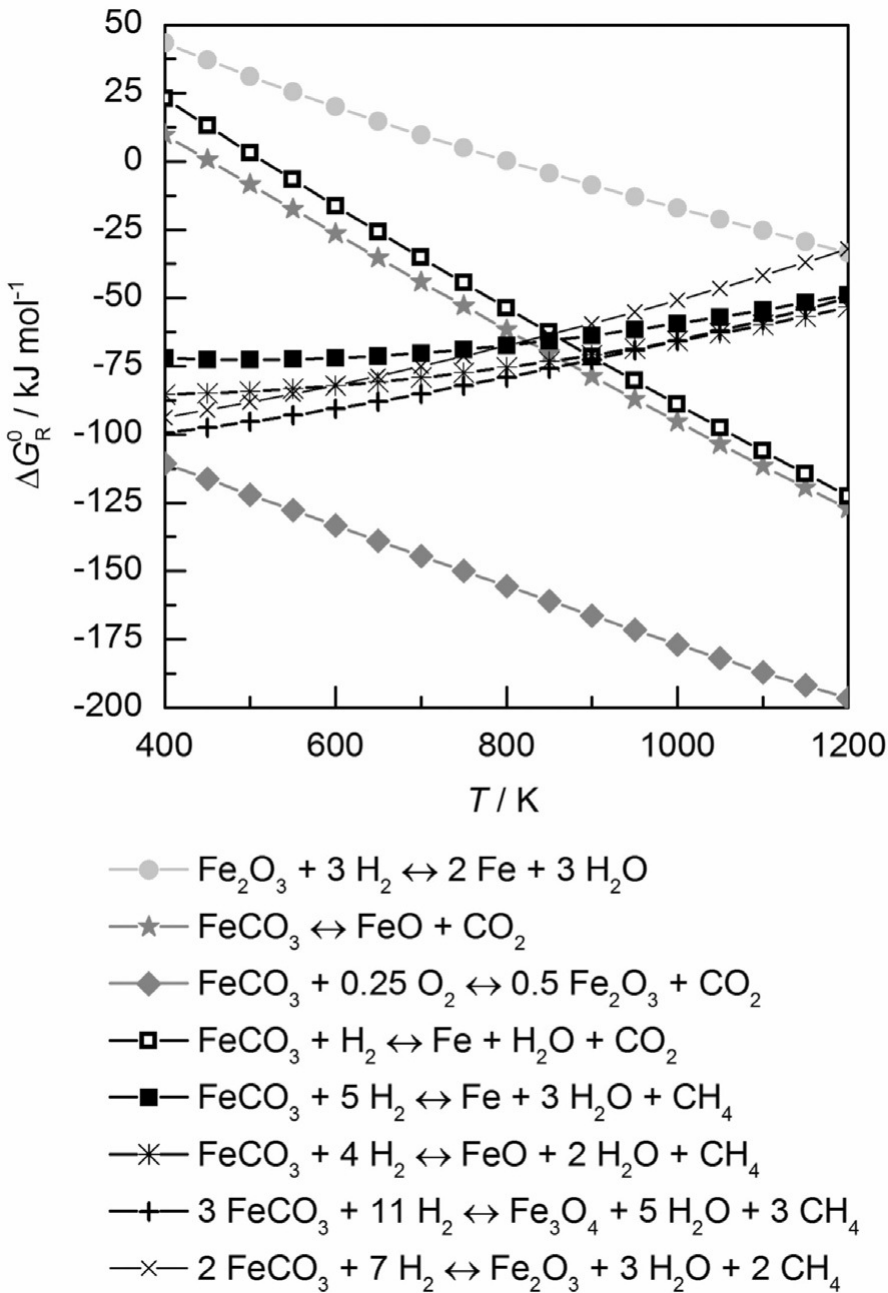
Standard free energies of reaction, Δ*G*_R_^0^, for decarboxylation of FeCO_3_ relative to hydrogenation of Fe_2_O_3_ between 400 and 1200 K at ambient pressure calculated with HSC Chemistry 8 software.

Figure 2 compares conventional beneficiation of FeCO_3_ under oxidizing conditions with hydrogenation of FeCO_3_. Both routes are thermodynamically favorable in the temperature range examined (400–1200 K), although reduction of hematite (Fe_2_O_3_) features significantly higher standard free energies of reaction.

Hydrogenation of the transition‐metal carbonates and MgCO_3_ to yield CH_4_ exhibit pronounced exothermic behavior (Δ*H*_R_^0^<−50 kJ mol^−1^).

## 4 Experimental Studies

Experimental studies on the hydrogenation of metal carbonates are scarce. Although first reports date back to the late 1960s, to the best of our knowledge, there are only 20 scientific publications from six research groups. Interestingly, hardly any cross reference exists between the studies.

Giardini and Salotti from the University of Georgia, USA, were the first to report on reactions between mineral calcite, dolomite, and siderite with pressurized hydrogen and the concomitant formation of hydrocarbons.^11^, ^45^, ^46^ The primary purpose of their study was to address geological issues, such as the formation of hydrocarbons from inorganic sources occurring in the earth's crust. A patent was filed on this topic.^47^ The experimental apparatus consisted of a 25 cm^3^ externally heated “cold seal”‐type vessel, in which the carbonate charge was enclosed in a platinum foil, and left unscaled and suspended in the heated part of the vessel. Before usage, the minerals were handpicked for impurities, crushed, and heated in 30 % hydrogen. The natural mineral deposit and the composition of the minerals were not stated.

From 1987, Reller and co‐workers from the University of Zürich, Switzerland, investigated the thermal reactivity of pure alkaline‐earth‐metal carbonates, 3d transition‐metal carbonates, and metal‐doped alkaline‐earth‐metal carbonates in pure and dilute hydrogen by thermogravimetric (TG) experiments.^10^, ^48^, ^49^

Two groups from Japan—one from the Tokyo Institute of Technology;^50^ the other from Kobe University^51^—investigated metal carbonate hydrogenation in the 1990s. Tsuneto et al. used NiCO_3_ and CoCO_3_ without additional catalysts and a series of metal carbonates doped with catalytically active metals, and hydrogenated them in a fixed‐bed flow reactor at atmospheric pressure.^50^ Formation of gaseous products was reported in μmol h^−1^ at a total gas flow of 9.6 cm^3^ min^−1^ and feed samples of 2 g after 0.5–1 h. To estimate the reaction rate, we calculated the hourly conversion of the respective carbonate to CH_4_ (*CtM* in % h^−1^), as depicted from Equation (7). Data listed in the table coincided only partially with the data given in the main text of their paper.^50^ In case of discrepancies, data listed tabularly were used herein.

(7) $$CtM=\;\left(1-\frac{n_{0,\;carbonate}-{\dot{n}}_{CH_{4}}}{n_{0,\;carbonate}}\right)\;\cdot 100$$

Yoshida et al. focused on CH_4_ formation from metal‐catalyzed CaCO_3_ hydrogenation, in comparison to hydrogenation of pure CaCO_3_.^51^ Experiments were carried out by means of the temperature‐programmed hydrogenation technique, and for isothermal kinetic runs two apparatuses were applied: a Cahn electrobalance and a closed circulation apparatus.

In 2009 and 2013, Jagadeesan et al. from the Jawaharlal Nehru Centre for Advanced Scientific Research, India, investigated CaCO_3_ and mixed‐metal/CaCO_3_ hydrogenation promoted with catalytically active metallic nanoparticles in a continuous‐flow, packed‐bed, stainless‐steel reactor and managed to directly convert the inorganic carbonates into C_1_–C_3_ hydrocarbons.^13^, ^52^

The effect of reaction temperature, pressure, and gas atmosphere on the reaction kinetics of mineral FeCO_3_, MgCO_3_, and CaMg(CO_3_)_2_ hydrogenation has recently become subject of detailed investigation by Baldauf‐Sommerbauer et al. from Graz University of Technology, Austria.^12^, ^53^, ^54^ Experiments were carried out in a thermobalance and a tubular reactor setup.

A detailed list of the abovementioned studies, starting with the most recent ones, is given in Table 1. The investigations can be divided into two groups: 1) hydrogenation of single‐metal carbonates without additional catalysts, applied either as mineral ore or in synthetic form; 2) mixed‐metal/metal carbonates, mainly synthetic, in which one of the metals, mostly transition metals, acts as an internal catalyst. In general, synthetic carbonates and mixed‐metal/metal carbonates were produced by precipitation and coprecipitation from aqueous solution of NaHCO_3_.

**Table 1:** Papers on metal carbonate hydrogenation dating back to the 1960s until the present (2018), ranked in chronological order.

Material component	Additional	Feed gas	Experimental	Sample		Flow rate	*T*	*P*	Ref.	Year
	catalyst	comp.	apparatus	size	mass [g]	[cm^3^ min^−1^]	[K]	[MPa]		
mineral MgCO_3_/CaMg(CO_3_)_2_^[a]^	no	H_2_ (90 vol %) N_2_ (10 vol %)	tubular reactor (T316 SS, *d=*2.5 cm, *l*=80 cm)	5–8 mm	121	500	768–808	amb 0.3, 0.8 (o.p)	54	2017
										
mineral MgCO_3_^[b]^	no	H_2_ (90 vol %) N_2_ (10 vol %)	tubular reactor (T316 SS, *d=*2.5 cm, *l*=80 cm)	5–8 mm	115	500	748, 763, 778	amb 0.3, 0.8 1.2 (o.p.)	99	2016
										
mineral FeCO_3_^[c]^	no	H_2_ (70 vol %) N_2_ (30 vol %)	thermobalance (HR=1.8, 3, 5, 10 K min^−1^)	100–200 μm	0.02	100	<1023	amb	12	2016
										
mineral FeCO_3_^[c]^	no	H_2_ (90 vol %) N_2_ (10 vol %)	tubular reactor (T316 SS, *d=*2.5 cm, *l*=80 cm)	0.5–1 mm	60	867	623, 648	amb	96	2016
										
CaCO_3_	Fe	H_2_	continuous‐flow, packed‐ bed reactor (SS)	n.a.	0.04	3	573–873	amb	13	2013
										
MeCa(CO_3_)_2_ (Me=Co, Ni, Fe; Me/Ca=1:1); Me^1^Me^2^Ca(CO_3_)_3_ (Me^1^Me^2^=CoNi, NiFe, FeCo; Me^1^/Me^2^/Ca=1:1:2)	no/yes, NPs of Co/CaO/CoO, Ni/CaO, CoNi/CaO/CoO Fe/CaO/Fe_3_O_4_, FeCo/CaO/CoO, NiFe/CaO	H_2_	tubular reactor (SS, *d=*0.6 cm, *l*=30 cm)	n.a.	0.05	3.5–8	823	amb	52	2009
										
CaCO_3_	no/yes, Pd, Ir (5 wt %)	H_2_ (<0.027 MPa)	temp.‐programmed hydrogenation (HR=3 K min^−1^), Cahn electrobalance (3.62 cm^3^), closed circul. apparatus (275 cm^3^)	n.a.	0.015, 0.1, 570	60	573–698	amb	51	1999
										
NiCO_3_ **⋅**Ni(OH)_2_ **⋅**4 H_2_O, CoCO_3_, Li_2_CO_3_, Na_2_CO_3_, K_2_CO_3_, MgCO_3_⋅Mg(OH)_2_ **⋅**5 H_2_O, BaCO_3_, CaCO_3_	no/yes; Ni, Fe, Co, Pd, Pt, Cu (2 wt %)	H_2_ (50 vol %) He (50 vol %)	fixed bed flow reactor	n.a.	2	9.6	473–673	amb	50	1992
										
MnCO_3_, FeCO_3_, CoCO_3_, NiCO_3_, ZnCO_3_, caustic 3d transition‐ metal carbonates (Cu, Ni, Zn, Cu/Ni, Cu/Co, Cu/Zn, Ni/Zn), MeMg(CO_3_)_2_ (Me=Fe, Co, Ni, Cu, Zn), Me^1^Me^2^Mg(CO_3_)_2_ (Me^1^Me^2^=CuZn, Ni/Zn, Cu/Ni)	no, influence of type of energy	H_2_ (5 % H_2_ in Ar)	TG/MS (HR=10 K min^−1^)		≈15×10^−3^	30	<900	amb	22	1992
										
Mg_5_(OH)_2_(CO_3_)_4_ **⋅**4 H_2_O	no/yes, Ni	H_2_	TG/DTA, TG/MS	n.a.	8.2×10^−3^	30	<900	amb	56	1991
										
MgCO_3_, CaCO_3_, SrCO_3_, BaCO_3_, FeCO_3_	no/yes; Ni, Ru, Rh for CaCO_3_	H_2_ (0.1 MPa)	TG/MS (HR=10 K min^−1^)	n.a.	≈15×10^−3^	30	<1200	amb	10, 55	1991 1989
										
CaCO_3_	Fe, Ni, Co, Cu, Ru, Rh, Pd, Ag	H_2_	TG/MS	n.a	10–12×10^−3^	30	<950	amb	49, 55	1990 1989
										
mineral MgCO_3_,^[d]^ mineral CaMg(CO_3_)_2_,^[e]^ mineral CaCO_3_^[f]^	Co, Ni, Cu (10 %) as (Mg,Me)(CO_3_)_2_, (Ca,Me)(CO_3_)_2_	H_2_ (0.1 MPa)	TG/MS (HR=10 K min^−1^)	n.a.	8.35–10.99×10^−3^	n.a.	<1000	amb	48, 55	1987 1989
										
mineral CaCO_3_, mineral CaMg(CO_3_)_2_, mineral FeCO_3_	metallic Ni, Pt, Cu, Ti, Mg, Fe; mixtures of Pd, Pt, Rh on Al_2_O_3_ and dried silica gel; activated Al_2_O_3,_ Fe_2_O_3_, Fe_3_O_4_, Cr_2_O_3_, CrO_3_, Kieselguhr mixtures; hydrous and anhydrous oxides, pyrolytic carbon	H_2_ (1.4–55 MPa)	25 cm^3^ cold seal, SS vessel	40–60 mesh	n.a.	DA	618–1143	amb	11, 46, 47	1968 1969 1971
										
mineral CaCO_3_	no	H_2_ (0.7–80 MPa)	25 cm^3^ cold seal, SS vessel	n.a.	n.a.	DA	693–1243	amb	45	1968

[a] Breitenau, Austria. [b] Eskişehir, Turkey. [c] Erzberg, Austria. [d] Ural, Russia. [e] Binn Valley, Switzerland. [f] Gonzen mine, Switzerland. amb=ambient pressure, o.p.=overpressure, n.a.=not available, TG=thermogravimetric experiments, MS=mass spectrometry, DTA=differential thermal analysis, SS=stainless steel, HR=heating rate, NPs=nanoparticles, DA=discontinuous operation.

### 4.1 Single‐metal carbonates

Hydrogenation of single‐metal carbonates includes the alkaline‐earth‐metal carbonates MgCO_3_, CaCO_3_, SrCO_3_, BaCO_3_, and CaMg(CO_3_)_2_ and the 3d transition‐metal carbonates MnCO_3_, FeCO_3_, CoCO_3_, NiCO_3_, and ZnCO_3_. Decomposition of the respective carbonates in a reducing hydrogen atmosphere occurs at lower temperatures than that of decomposition under inert or oxidizing conditions.

#### 4.1.1 Alkaline‐earth metals

##### MgCO_3_, CaCO_3_, BaCO_3_, and SrCO_3_

Reller et al.^48^ and Padeste^55^ performed TG measurements with finely ground MgCO_3_ and CaCO_3_ mineral, and synthetic SrCO_3_ and BaCO_3_ at atmospheric pressure. Equal amounts of CO_2_ and CO, together with H_2_O, were formed as gaseous products from MgCO_3_. In the case of CaCO_3_, the main volatile compound was CO (CO/CO_2_≈10:1) from which a change in the degradation mechanism was concluded. The concomitant formation of CO, in addition to CO_2_ and H_2_O, was dedicated to the reduction of CO_2_ through the reverse water‐gas shift reaction [Eq. (6)]. The amount of CO increases with increasing atomic mass of the alkaline‐earth‐metal cation due to higher decarboxylation temperatures (*T* ${}_{\mathrm{MgCO}{}_{3}}$ <800 K, *T* ${}_{\mathrm{CaCO}{}_{3}}$ ≈900 K) and the endothermic nature of the reverse water‐gas shift reaction, which shifts the equilibrium composition towards the product CO at higher temperatures. The reaction temperature for decarboxylation in hydrogen was lowered by at least 150 K compared with the analogous reaction in a nitrogen atmosphere. In a hydrogen atmosphere, all four alkaline‐earth‐metal carbonates fully degraded below temperatures of 1200 K into their respective oxides MgO, CaO, SrO, and BaO. They formed as solid conglomerates of microcrystalline domains with diameters of 10–20 nm and showed a pronounced reactivity towards the respective hydroxides and towards recarbonation.^48^ If synthetic CaCO_3_ crystals were degraded in nitrogen and hydrogen atmospheres, distinct destruction in nitrogen occurred, which suggested that diffusion of H_2_ into the carbonate, where it directly reacted with fixed CO_2_, and reverse diffusion of formed CO and H_2_O proceeded faster than that of CO_2_ diffusion.^10^, ^48^

Kinetic studies with CaCO_3_ were performed by Yoshida et al. in a closed circulation apparatus at a fixed H_2_ pressure of 0.13×10^5^ Pa and 748 K.^51^ The reaction was of half‐order with respect to H_2_ with an activation energy of 236 kJ mol^−1^. Initial reaction rates did not differ for varying CaCO_3_ amounts, which was explained by decomposition of CaCO_3_ and hydrogenation of released CO_2_ to CO. The lowest temperature at which hydrogenation occurred was 700 K. At temperatures of 700–773 K, only CO formed. Above 773 K, CO and CO_2_ formed. The formation of the two gaseous products differed over the course of time. Whereas CO pressure steadily increased with time, CO_2_ pressure abruptly reached a maximum value of 22.7 Pa and did not change subsequently.

Baldauf‐Sommerbauer et al. examined the effect of reaction temperature (748–778 K) and pressure (ambient to 1.2 MPa overpressure) on the reductive calcination of magnesite (5–8 mm) in a fixed‐bed tubular reactor with 70 % hydrogen and nitrogen.^53^ MgCO_3_ conversion rose with increasing reaction temperature (e.g., from 45 % at 748 K to 96 % at 778 K). Under isothermal conditions, the conversion decreased with increasing pressure (e.g., at 763 K from 76 % at ambient pressure to 67 % at 1.2 MPa overpressure). In contrast to the findings of Reller et al.,^48^ CH_4_ was found in addition to CO_2_, CO, and H_2_O as gaseous products [Eq. (8)].

(8) $$\left(a+b+c\right)\;\mathrm{MgCO}{}_{3}+\left(b+4\;c\right)\;H{}_{2}\to \left(a+b+c\right)\;\mathrm{MgO}+a\;\mathrm{CO}{}_{2}+b\;\mathrm{CO}+c\;\mathrm{CH}{}_{4}+\left(b+2\;c\right)\;H{}_{2}O$$

Low temperature and elevated pressure facilitated CH_4_ formation. Moderate to high temperature and low pressure facilitated CO formation. The CH_4_ yield was 38.6 % after 20 % MgCO_3_ conversion at 748 K and 1.2 MPa overpressure. With respect to the reaction mechanism, decreasing CH_4_ formation with increasing magnesite conversion indicated a dependency on the amount of MgCO_3_. Baldauf‐Sommerbauer et al. related the increase of CO concentration with rising MgCO_3_ conversion to the amount of MgO. To scrutinize this interpretation, they examined reductively calcined MgO for its catalytic properties for CO_2_ conversion with H_2_. At ambient pressure and 0.3 MPa overpressure, only CO formed. At 0.8 MPa overpressure, traces of CH_4_ occurred in addition to the major product CO. These findings revealed significant reverse water‐gas shift activity of reductively calcined MgO, but no CH_4_ formation. CH_4_ formation during reductive calcination of magnesite seemingly proceeds through a different mechanism.

Giardini and Salotti reported the formation of CH_4_ and ethane (C_2_H_6_) through heating (693–1243 K) of calcite and dolomite under a pressurized hydrogen atmosphere (0.7–80 MPa H_2_); however, it was not comprehensible whether the pure minerals were used or if the reaction was catalytically accelerated.^11^, ^45^, ^46^, ^47^ In their main publication,^11^ they stated that metallic Ni, Pt, Cu, Ti, Mg, and Fe; commercial mixtures of 0.5 % Pd, Pt, Rh on alumina and dried silica gel; activated alumina; hematite, magnetite; chromic oxide; chromium trioxide; and Kieselguhr mixtures were added, but no precise information was given on the type of catalyst for individual experimental data. The catalyst admixture was neither mentioned in the first publication^46^ nor in the patent application.^47^ Apparently, none of the catalytically active materials had a discernible effect on the rate of reaction. The reaction kinetics were thus expected to fit the uncatalyzed hydrogenation of calcite. Due to high initial hydrogen concentrations relative to calcite, the reaction kinetics simplified to pseudo‐first‐order kinetics, with an activation energy of 75 kJ mol^−1^ at 14 MPa.^45^ Hydrogenation of calcite started at 773 K. It was primarily dependent on temperature and secondarily dependent on pressure and time and proceeded in a crystallographically anisotropic manner. As solid products, CaO, Ca(OH)_2_, graphite (C), and a black residue, possibly solid hydrocarbons, formed. At higher temperatures, CaO was the principal solid product. CH_4_ and H_2_O were ubiquitous gaseous products, whereas C_2_H_6_ and CO appeared under certain specific conditions. CO_2_ was never detected in remarkable amounts (>0.01 %). CO_2_, if formed, immediately converted into CH_4_, CO, and H_2_O. The maximum CH_4_ content (on a dry basis) was 3.2 % (and 96.75 % H_2_) at 988 K, 61 MPa, and 16 h. The reaction was dependent on the type of surface because powdery CaCO_3_ reacted faster than a single rhomb of equal weight.

##### Mg_5_(OH)_2_(CO_3_)_4_⋅4 H_2_O

Hydrogenation of hydromagnesite [Mg_5_(OH)_2_(CO_3_)_4_⋅4 H_2_O] to MgO occurred in two steps and finished at temperatures lower than 750 K. The first step corresponded to loss of water. In the second step, CO_2_ released from Mg_5_(OH)_2_(CO_3_)_4_ and yielded MgO as the final solid product.^56^

##### CaMg(CO_3_)_2_

The effect of reaction temperature (793 K–1108 K) and initial hydrogen pressure (14–34 MPa) on the hydrogenation of dolomite (40–60 mesh) was investigated by Giardini and Salotti.^46^ As for calcite, there was no clear information on the catalyst admixture. We assume that the main findings apply to the hydrogenation of dolomite without additional catalysts. Solid products included CaCO_3_, Ca(OH)_2_, CaO, noncrystalline Mg(OH)_2_, graphite, and a “soot‐like” material; gaseous products CH_4_, C_2_H_6_, CO, and CO_2_. H_2_O was the oxygenated product in all experiments. A two‐step reaction mechanism was proposed in which noncrystalline Mg(OH)_2_ or MgO formed [Eq. (9)]. At 34 MPa, the reaction started at 793 K.

(9) $$\mathrm{CaMg}\left(\mathrm{CO}{}_{3}\right){}_{2}+4\;H{}_{2}\to \mathrm{CaCO}{}_{3}+\mathrm{Mg}\left(\mathrm{OH}\right){}_{2}+\mathrm{CH}{}_{4}+H{}_{2}O$$

Reller et al. found that the decarboxylation temperature of dolomite and the ratio of the gaseous products CO and CO_2_ lay between the corresponding values of the two pure carbonates CaCO_3_ and MgCO_3_.^48^

Baldauf‐Sommerbauer et al. suggested the hydrogenation of mixed magnesite/dolomite (1:1 mol/mol) for the synthesis of CO.^54^ TG measurements showed two decomposition steps. Compared with the reaction in nitrogen, a hydrogen atmosphere leads to a decrease of the decomposition temperature of 60 K for the first step and 100 K for the second step. In the first step, the decomposition of concomitant MgCO_3_, FeCO_3_, and MnCO_3_ to the respective bivalent oxides and CO_2_ was observed. Then CaMg(CO_3_)_2_ decomposed into CaO, MgO, and CO_2_. Reductive calcination experiments in a tubular reactor indicated a sequential mechanism of calcination followed by hydrogenation of CO_2_. CH_4_ was only formed in traces, even at elevated pressure. It is assumed that CH_4_ formation was kinetically hindered. A CO yield of 61–73 % was achieved for partial reductive calcination of the magnesite content below 813 K. An increase of pressure did not affect the formation of CO, but caused a slight retardation of the reaction.

#### 4.1.2 Transition‐metal carbonates

The minerals MnCO_3_ (rhodochrosite) and FeCO_3_ (siderite), and synthesized CoCO_3_, NiCO_3_, and ZnCO_3_ were investigated. Synthesis requires hydrothermal conditions at high pressure. According to the size of their cations, the 3d transition‐metal carbonates crystallize in a trigonal calcite‐type structure.^22^, ^57^

As postulated by Emmenegger^22^ from hydrogenation experiments in pure and dilute hydrogen (5 % H_2_ in Ar), the partial hydrogen pressure decisively influences the hydrogenation reaction. Different solid products—transition‐metal oxides and elemental transition metals arise—depending on the selected atmosphere, and morphological features may be controlled. In hydrogen, decarboxylation temperatures drop, in comparison to the respective reaction under an inert atmosphere. Dilute hydrogen results in a lower temperature drop. The degradation temperature drops with decreasing radii of the transition‐metal cations: Mn^2+^ (0.8 Å)>Fe^2+^ (0.76 Å)>Co^2+^ (0.74 Å)>Ni^2+^ (0.72 Å)>Mg^2+^ (0.65 Å). The ratio of gaseous products CO_2_, CO, CH_4_, higher hydrocarbons, and H_2_O varies, depending on the transition‐metal species. According to Reller et al., CO_2_ is released during transition‐metal carbonate hydrogenation, and further catalytically hydrogenated.^10^ In the course of decarboxylation, the catalysts Me or Me/MeO form in situ. Fe, Co, and Ni act as efficient hydrogenation catalysts.

##### MnCO_3_

In pure and dilute (5 % H_2_ in Ar) hydrogen, the mineral MnCO_3_ decarboxylated to MnO without changing its oxidation number at 643 K. As gaseous products, traces of unconverted CO_2_, CO, and H_2_O formed. Consequently, manganese efficiently catalyzed the reverse water‐gas shift reaction. In dilute hydrogen, this reaction was retarded. After a period of induction, released CO_2_ was further converted into CO and H_2_O. Compared with pure hydrogen, the chemical equilibrium barely lay on the side of the product.^22^

##### FeCO_3_

Salotti and Giardini first studied the hydrogenation of siderite (40–60 mesh) at reaction temperatures of 618 to 878 K and initial partial hydrogen pressures of 1.4 to 34 MPa (H_2_).^46^ Because there was no precise information on the catalyst admixtures reported, we assumed that the main findings applied to siderite only. At temperatures above 728 K, the solid products consisted of elemental Fe and wüstite (FeO). At lower temperatures (673 K and 14 MPa H_2_), magnetite (Fe_3_O_4_) formed. From minute and rare flecks, Giardini and Salotti assumed that also graphite was formed.^11^ Wüstite was the primary alteration product [Eq. (10)]. Further reduction of wüstite to elemental Fe [Eq. (11)] or oxidation of wüstite to magnetite [Eq. (12)] depended on the reaction temperature and the ultimate H_2_O/H_2_ ratio. A low reaction temperature and dry hydrogen need to be provided to effect reduction.

(10) $$\mathrm{FeCO}{}_{3}+5\;H{}_{2}↔\mathrm{FeO}+\mathrm{CH}{}_{4}+2\;H{}_{2}O$$

(11) $$H{}_{2}+\mathrm{FeO}↔\mathrm{Fe}+H{}_{2}O$$

(12) $$H{}_{2}O+3\;\mathrm{FeO}↔\mathrm{Fe}{}_{3}O{}_{4}+H{}_{2}$$

CH_4_ and H_2_O were ubiquitous gaseous products, whereas CO, CO_2_, and the higher hydrocarbons ethane (C_2_H_6_), propane (C_3_H_6_), and butane (C_4_H_10_) were present over a limited temperature and pressure range. An inverse relationship existed between the temperature and the length of the hydrocarbon chain, meaning that a lower initial reaction temperature results in a more complex hydrocarbon species. At 673 K (14 MPa H_2_), the gaseous product on a dry basis contained 4.45 mol % CH_4_, 0.28 mol % ethane, 0.01 mol % propane, and 0.03 mol % butane. At 728 K (1.4 MPa H_2_), the concentration of CH_4_ slightly decreased (4.34 mol %), whereas the concentrations of higher hydrocarbons increased (0.42 mol % ethane, 0.23 mol % propane, 0.05 mol % butane). At 878 K and 14 MPa H_2_, only CH_4_ was detected. CO_2_ only formed at 798 K (34 MPa H_2_). Giardini and Salotti explained this by the thermal stability of the hydrocarbons.^11^ The reaction temperature for siderite hydrogenation is low enough to ensure thermal stability of ethane, propane, and butane. They concluded that higher hydrocarbons formed directly through a reaction on the mineral surface, rather than in a subsequent reaction between released gases and hydrogen.

This conclusion contradicts the findings of Reller et al., who explained the formation of CO and CH_4_ by in situ formation of catalytically active transition‐metal species.^10^ Emmenegger compared the hydrogenation of siderite in pure and dilute hydrogen (5 % H_2_ in Ar).^22^ In pure hydrogen, the formation of mainly elemental Fe together with FeO as solid products was observed. At elevated temperature above 823 K, decomposition was slow and not yet finished at 973 K. The gaseous products CH_4_, H_2_O, and CO were formed. Higher hydrocarbons were not found. CO_2_ was also released. The reaction pathways in Equations (13), (14) were postulated for siderite hydrogenation. Because Fe was not the main product, according to Equation (14) reduction occurred only partially. The reverse water‐gas shift reaction for the reduction of CO_2_ to CO was reported.

(13) $$\mathrm{FeCO}{}_{3}↔\mathrm{FeO}+\mathrm{CO}{}_{2}$$

(14) $$\mathrm{FeO}+H{}_{2}↔\mathrm{Fe}+H{}_{2}O$$

In dilute hydrogen, CH_4_ did not form. The major amount of CO_2_ released from siderite [Eq. (13)] was not converted, but formed the main product of the product gas. Reduction of intermediate FeO was incomplete and only minor amounts of Fe were formed. In addition to the distinct difference in product composition between conversion in pure and dilute hydrogen atmospheres, solid products also differed in morphology. In pure hydrogen, the solid product contained a high amount of crystalline parts, whereas the product in dilute hydrogen featured a higher amount of fine particles. The conversion temperature significantly reduced in pure hydrogen (603 K), with respect to an inert helium atmosphere (653 K). In dilute hydrogen, decarboxylation started at 623 K.

Baldauf‐Sommerbauer et al. suggested the hydrogenation of siderite as a means for sustainable iron production.^12^ They performed kinetic computations to study the reaction kinetics of iron formation, and considered the concomitant decomposition of the accessory matrix carbonates of calcium, magnesium, and manganese The original mineral consisted of three main carbonate components of siderite FeCO_3_ with partial Mg and Mn substitution, ankerite (Ca_*a*_Fe_*b*_Mg_*c*_Mn_*d*_)CO_3_, and dolomite CaMg(CO_3_)_2_. Potassium, aluminum, and silicon existed in the form of muscovite KAl_2_(AlSi_3_O_10_)(OH)_2_, whereas a major part of the silicon was quartz (SiO_2_). A concentrated siderite specimen (size fraction 100–200 μm) was used for kinetic analysis in a thermobalance. During conversion under a hydrogen atmosphere, the FeCO_3_ content of mineral siderite was converted into elemental Fe [(79±2) wt %; Eq. (15)]. From calcium, magnesium, and manganese carbonates, the respective oxides formed [Eq. (16)].

(15) $$\mathrm{FeCO}{}_{3}+H{}_{2}\to \mathrm{Fe}+H{}_{2}O+\mathrm{CO}{}_{2}$$

(16) $$\left(\mathrm{Ca}{}_{x}\mathrm{Mg}{}_{y}\mathrm{Mn}{}_{z}\right)\mathrm{CO}{}_{3}\to x\;\mathrm{CaO}+y\;\mathrm{MgO}+z\;\mathrm{MnO}+\mathrm{CO}{}_{2}$$

The model‐free kinetic analysis, according to the Ozawa–Flynn–Wall,^58^, ^59^ Kissinger–Akahira–Sunose,^60^ and Friedman^61^ approaches, gave a parallel kinetic model. With multivariate nonlinear regression, the kinetic parameters (*E*_a_=151.8 kJ mol^−1^, log 10(*A*)=8.751 s^−1^) were determined. A two‐dimensional Avrami–Erofeev model A2 was applicable for the conversion of FeCO_3_ into Fe. Therefore, a temperature‐controlled nucleation and diffusional growth mechanism was suggested. For the concomitant formation of CaO, MgO, and MnO, multiparameter autocatalysis models were used without applying multistep kinetics. At 723 K, more than 95 % conversion within less than 60 min reaction time was observed. Increasing temperature leads to a faster reaction, but a lower yield of elemental Fe.

##### CoCO_3_ and NiCO_3_

Hydrogenation of synthetic CoCO_3_ and NiCO_3_ was investigated by Emmenegger^22^ and Tsuneto et al.^50^ Tsuneto et al. hydrogenated commercial transition‐metal powders of CoCO_3_ and NiCO_3_⋅Ni(OH)_2_⋅4 H_2_O in a fixed‐bed flow reactor at atmospheric pressure and 473 K.^50^ In both cases, CH_4_ formed after a period of induction, in which only CO_2_ evolved through thermal decomposition. In the case of CoCO_3_, a maximum methane formation rate of 97 μmol h^−1^ was achieved after 55 h. With NiCO_3_, the methane formation rate reached a maximum value (72 μmol h^−1^) after 7 h and hydrogen (12 mmol h^−1^) was consumed completely. At 523 K, CH_4_ and CO_2_ formed promptly. The formation of metallic Co and Ni, together with the period of induction for methane formation, suggests that Ni and Co act as hydrogenation catalysts for CO_2_.

Emmenegger performed TG experiments with synthetic CoCO_3_ and NiCO_3_ in helium and pure and dilute hydrogen (5 % H_2_ in Ar).^22^ The temperature at which carbonate degradation started dropped from 603 K in helium to 543 K in pure hydrogen for CoCO_3_. For NiCO_3_, a similar temperature dependency of conversion, with 623 K in helium, 548 K in dilute hydrogen, and 513 K in pure hydrogen, was observed. Under a hydrogen atmosphere (pure and dilute), elemental Co and Ni formed as solid products. Similar behavior between CoCO_3_ and NiCO_3_ was also visible, in terms of the gaseous product stream. The main gaseous products CH_4_ and H_2_O formed. The byproducts CO_2_ and barely any CO were detected. The formation of gaseous products proceeded simultaneously. CO_2_ fully converted after a certain period of induction, which indicated that cobalt and nickel catalyzed the hydrogenation of CO_2_ into CH_4_. Conversion differed with respect to the rate of reaction in dilute hydrogen: at the beginning, the conversion of NiCO_3_ was slower than that of CoCO_3_. In both cases, the catalytic activity for CO_2_ hydrogenation dropped drastically and the main gaseous products were CO_2_ and H_2_O [Eqs. (13) and (14) for CoCO_3_ and NiCO_3_] and only minor amounts of CO. These reactions seemed to occur simultaneously because CO_2_ and H_2_O formed concurrently. From CoCO_3_ conversion, barely any CH_4_ formed, but still significant amounts of CH_4_ arose from NiCO_3_. Consequently, Ni was confirmed to be a more efficient catalyst in a dilute hydrogen atmosphere than Co.

##### ZnCO_3_

Contrary to other 3d transition‐metal carbonates, Emmenegger found that decomposition temperatures of ZnCO_3_ increased from 583 K in pure hydrogen to 628 K in dilute hydrogen (5 % H_2_ in Ar).^22^ Due to a lower hydrogen concentration, the equilibrium composition of the reverse water‐gas shift reaction preferably consists of H_2_ and CO_2_. Zinc did not show any catalytic activity for the reduction of evolved CO_2_. The composition of the gaseous product mixture (CO_2_, CO, and H_2_O) resembled that expected for the water‐gas equilibrium.

##### Caustic 3d transition‐metal carbonates

Emmenegger investigated the hydrogenation of caustic 3d transition‐metal carbonates of the type Me_*y*+*x*/2_(OH)_*x*_(CO_3_)_*y*_⋅*z* H_2_O (Me=Cu, Ni, Zn), combinations thereof (Cu/Ni, Cu/Co, Cu/Zn, Ni/Zn), and mixed caustic transition‐metal/Mg carbonates (MeMg(CO_3_)_2_ with Me=Fe, Co, Ni, Cu, Zn; Me_1_Me_2_Mg(CO_3_)_2_ with Me_1_Me_2_=CuZn, Ni/Zn, Cu/Ni).^22^ Decomposition temperatures were lower than those of the respective neutral carbonates. Loss of crystal water and condensation of OH^−^ with caustic carbonates was observed. Higher water concentration shifts the equilibrium composition of potential CO_2_ hydrogenation to the reactant side. Consequently, all caustic metal carbonates show low catalytic hydrogenation activity compared with that of the respective neutral carbonates. Mixed transition‐metal/Mg carbonate systems show pronounced catalytic activity, which highlights the effect of the noncatalytic support material MgO. MgO forms fine particles that give access to high dispersion of the transition metals or alloys.^22^

### 4.2 Main‐group metal carbonates combined with transition metals

An admixture of transition metals to main‐group metal carbonates opens up a new pathway in metal carbonate hydrogenation: because many transition metals catalyze hydrogenation reactions, CO_2_ evolved from the carbonate is converted into CO, CH_4_, or higher hydrocarbons C_*x*_H_*y*_ and C_*x*_H_*y*_O_*z*_. The catalytically active transition‐metal species is formed in situ during the decomposition reaction. A wide range of transition metals (Fe, Ni, Co, Cu from the 3d group; Ru, Rh, Pd, Ir, Ag from the 4d group) was used for doping of various main‐group metal carbonates, such as Li_2_CO_3_, Na_2_CO_3_, K_2_CO_3_, CaCO_3_, MgCO_3_, SrCO_3_, 4 MgCO_3_⋅Mg(OH)_2_⋅5 H_2_O, and BaCO_3_. Mixed alkaline‐earth‐metal/transition‐metal carbonates of the type MeCa(CO_3_)_2_ and Me_1_Me_2_(CO_3_)_3_ were also investigated.

In general, two promising effects take place. First, the decarboxylation temperature of the alkaline and alkaline‐earth‐metal carbonates drops. Second, different gaseous compounds evolve during decomposition due to the catalytic activity of the transition‐metal species. The product composition depends on the transition metal in the carbonate.

#### 4.2.1 Alkaline‐metal carbonates

Tsuneto et al. hydrogenated nickel‐doped (2 wt %) Li_2_CO_3_, Na_2_CO_3_, and K_2_CO_3_ at 400 K and atmospheric pressure.^50^ Nickel powder was mechanically mixed with the carbonate sample. CO_2_ was not detected after 1 h from Li_2_CO_3_ and Na_2_CO_3_ decomposition, according to thermodynamics. Traces of CO_2_ were detected from K_2_CO_3_ (<2 μmol h^−1^ at a total gas flow rate of 9.6 cm^3^ min^−1^). With all three carbonates, minor amounts of CH_4_ were formed (Li_2_CO_3_: 1.3 μmol h^−1^, *CtM*=0.005 %, Na_2_CO_3_: 2.5 μmol h^−1^, CtM=0.013 %, K_2_CO_3_: 1.8 μmol h^−1^, *CtM*=0.012 %).

#### 4.2.2 Alkaline‐earth‐metal carbonates

Hydrogenation of calcite and dolomite minerals at elevated temperatures (calcite: 693–1143 K, dolomite: 793–1108 K) and elevated pressure (calcite: 1.4–55 MPa H_2_, dolomite: 14–34 MPa H_2_) was investigated by Giardini and Salotti.^11^ In their report, it is ambiguous to which experimental runs metallic Ni, Pt, Cu, Ti, Mg, and Fe, or commercial mixtures of 0.5 % Pd, Pt, and Rh on alumina and dried silica gel, activated alumina, hematite, magnetite, chromic oxide, chromium trioxide, Kieselguhr mixtures, hydrous and anhydrous oxides, and pyrolytic carbon were added as potential catalysts. None of the catalytically active materials had a discernible effect on the rate of reaction. The metals Pt, Fe, and Ni catalytically promoted pyrolytic dissociation of formed CH_4_ [Eq. (17)]. For calcite, they reported the formation of CaO [Eq. (18)]; Ca(OH)_2_ [Eq. (19)]; graphite; and, at temperatures above 973 K, carbon soot‐like material, which was seemingly amorphous carbon formed through thermal dissociation of CH_4_ [Eq. (17)]. Gaseous products were CH_4_, C_2_H_6_, CO, CO_2_, and H_2_O. Below the dehydration temperature, Ca(OH)_2_ was the stable solid product.

(17) $$\mathrm{CH}{}_{4}↔C+2\;H{}_{2}$$

(18) $$\mathrm{CaCO}{}_{3}+4\;H{}_{2}↔\mathrm{CaO}+\mathrm{CH}{}_{4}+2\;H{}_{2}O$$

(19) $$\mathrm{CaCO}{}_{3}+4\;H{}_{2}↔\mathrm{Ca}\left(\mathrm{OH}\right){}_{2}+\mathrm{CH}{}_{4}+H{}_{2}O$$

They assumed that CH_4_, and its higher homologues, if thermally stable under the reaction conditions, formed directly through methanation of calcite, rather than through reactions between H_2_, CO_2_, and CO. CO_2_ and CO were only detected at low pressure (1.4 MPa) and high reaction temperature (973 K).

Reller et al. investigated the effects of Co, Ni, and Cu doping (10 %) on the hydrogenation of MgCO_3_ and CaCO_3_.^48^ Mixed alkaline‐earth‐metal/transition‐metal carbonates, Mg−Me and Ca−Me carbonates, were prepared by coprecipitation with sodium carbonate from the respective nitrate solutions. In a second study, the effect of coprecipitated Ni, Ru, and Rh was reported.^10^ The decarboxylation temperatures dropped in the range of 200 K to, at most, 400 K in the case of Ni, compared with the decarboxylation of the pure carbonates in a nonreducing atmosphere. Mixtures of CO and CO_2_ were released if Cu was used as a coprecipitate. In the case of Co, predominantly CH_4_ formed together with minor amounts of CO. The formation of CO_2_ was negligible. With Ni on MgCO_3_ and CaCO_3_, more than 90 % of the gaseous product was CH_4_. The formation of C_*x*_H_*y*_O_*z*_ species from CaCO_3_ in H_2_ was not detected at atmospheric pressure (*p=*0.1 MPa). The solid products consisted of a mixture of microcrystalline alkaline‐earth‐metal oxides and elemental transition metals. Reller et al. confirmed that the formed solid products acted as effective catalysts for the partial reduction of CO_2_ to CO or direct conversion of CO_2_ into CH_4_. For Ni−Ca carbonate systems, the activity of the system was explained by the high dispersion of the catalytically active transition metal in the CaCO_3_ matrix.^48^ Consequently, the combination of transition‐metal carbonates with alkaline‐earth‐metal carbonates improves the catalytic activity of the in situ formed transition‐metal species if an appropriate dispersion or active surface area is generated during decarboxylation; an effect that cannot be accomplished with pure transition‐metal carbonates only.

Padeste et al. published a detailed study comparing the influence of the 3d transition metals Fe, Ni, Co, and Cu and the 4d transition metals Ru, Rh, Pd, and Ag on the thermal decomposition of CaCO_3_ in hydrogen.^49^ The mixed‐metal carbonates were produced by coprecipitation. Whereas 3d metal carbonates, except for FeCO_3_, normally precipitate as caustic carbonates (hydroxocarbonates) from aqueous solutions, ions of the 4d metals Rh, Ru, and Pd precipitate as oxides or oxide hydrates and Ag predominantly forms the simple carbonate Ag_2_CO_3_. Samples from NiCO_3_/CaCO_3_ and CoCO_3_/CaCO_3_ showed that two‐phase systems formed, even at transition‐metal carbonate concentrations of 5 %, rather than replacing large amounts of Ca^2+^ by transition metals with similar ionic radii. TG measurements showed that thermal decomposition in hydrogen proceeded in two steps: First, decomposition and reduction of the transition‐metal carbonate below temperatures of 600–700 K with H_2_O evolution. Second, CaCO_3_ decomposition at temperatures above 600–700 K. H_2_O evolution might result from loss of coprecipitated water, decomposition of hydroxides to oxides [Eq. (20)], reduction of CO_2_ [Eqs. (21), (22)], and reduction of the metal oxide formed [Eq. (23)].

(20) $$2\;\mathrm{OH}{}^{-}\to O{}^{2-}+H{}_{2}O$$

(21) $$\mathrm{CO}{}_{2}+H{}_{2}\to \mathrm{CO}+H{}_{2}O$$

(22) $$\mathrm{CO}{}_{2}+4\;H{}_{2}\to \mathrm{CH}{}_{4}+2\;H{}_{2}O$$

(23) $$\mathrm{MeO}+H{}_{2}\to \mathrm{Me}+H{}_{2}O$$

The reaction in Equation (23) was observed for all transition‐metal carbonates. CO formation [Eq. (21)] did not occur in this temperature range and CH_4_ formation [Eq. (22)] was only observed for Ni and Co. During the CaCO_3_ decomposition step, CO_2_ [Eq. (24)], CO [Eq. (25)], and CH_4_ [Eq. (26)] formed as volatile carbon products.

(24) $$\mathrm{CaCO}{}_{3}\to \mathrm{CaO}+\mathrm{CO}{}_{2}$$

(25) $$\mathrm{CaCO}{}_{3}+H{}_{2}\to \mathrm{CaO}+\mathrm{CO}+H{}_{2}O$$

(26) $$\mathrm{CaCO}{}_{3}+4\;H{}_{2}\to \mathrm{CaO}+\mathrm{CH}{}_{4}+2\;H{}_{2}O$$

Most CO_2_ reduces to CO and CH_4_. A correlation between the decomposition temperature and the distribution of the volatile products based on the thermodynamics of the different reactions was stated: carbonates that evolve predominantly CH_4_ decompose at the lowest temperature, whereas carbonates that release CO as the main gaseous compound have the highest decomposition temperature, which resembles the behavior of pure CaCO_3_. Whereas Fe, Cu, and Ag showed little influence (*T*_decomposition_=730–880 K, CH_4_>1 %, CO>90 %) on the thermal decomposition of CaCO_3_ in hydrogen, Co and Pd had a medium effect (*T*_decomposition_=680–850 K, 20 %<CH_4_<70 %), and Ni, Ru, and Rh had a pronounced effect (*T*_decomposition_=620–780 K, CH_4_>95 %).

Hydrogenation of 4 MgCO_3_⋅Mg(OH)_2_⋅5 H_2_O (at 573 K), CaCO_3_ (at 473, 573 and 673 K), and BaCO_3_ (at 673 K) in the presence of Ni powder in a fixed‐bed reactor was compared by Tsuneto et al.^50^ MgCO_3_ was readily hydrogenated to form a considerable amount of CH_4_ (1600 μmol h^−1^) with an hourly conversion to CH_4_ of 38.9 % and minor amounts of CO_2_ (290 μmol h^−1^). Contrary to MgCO_3_, no CO_2_ was released from CaCO_3_ and BaCO_3_ under the conditions investigated. CH_4_ formed in minor amounts from BaCO_3_ (1.8 μmol h^−1^, *CtM*: 0.018 %) and higher amounts from CaCO_3_ (95 μmol h^−1^, *CtM*: 0.475 %) at 673 K. The effect of reaction temperature was also investigated for Ni‐doped CaCO_3_. Although CH_4_ formation was not suppressed at temperatures as low as 473 K, the rate of CH_4_ formation decreased with decreasing temperature (473 K: 3.7 μmol h^−1^, *CtM*: 0.019 %; 573 K: 8.5 μmol h^−1^, *CtM*: 0.053 %; 673 K: 95 μmol h^−1^, *CtM*: 0.475 %). To connote, a remarkable shift of 400–500 K towards lower hydrogenation temperatures occurred in comparison to undoped CaCO_3_ (1172 K). Similar to the work of Padeste et al.,^49^ Tsuneto et al.^50^ investigated the effect of the transition metals Fe, Co, Ni, Pd, Pt, and Cu on CaCO_3_ hydrogenation at 673 K. The order of the catalytic activity was Ni>Co>Pt>Fe>Cu>Pd. This sequence confirms the results of Padeste et al.,^49^ apart from Pd, which was attributed medium influence in the former study. Long‐term hydrogenation was investigated for Ni‐doped CaCO_3_ at 673 K. The CH_4_ formation rate was constant for 7 days. Within 15 days, a total of 95 % of CaCO_3_ was converted. As solid products, CaO and Ca(OH)_2_ formed according to Equations (26) and (27).

(27) $$\mathrm{CaCO}{}_{3}+4\;H{}_{2}\to \mathrm{Ca}\left(\mathrm{OH}\right){}_{2}+\mathrm{CH}{}_{4}+H{}_{2}O$$

Because the rate of CH_4_ formation was greater than that of CO_2_ evolution, Tsuneto et al. concluded that direct CaCO_3_ hydrogenation occurred on the CaCO_3_ surface by hydrogen spillover from a Ni surface, rather than thermal decomposition of CaCO_3_ followed by hydrogenation of released CO_2_.^50^

Yoshida et al. studied methane formation through the hydrogenation of CaCO_3_ catalyzed by Pd and Ir (5 wt %) over a temperature range of 573–698 K.^51^ Temperature‐programmed hydrogenation data revealed high‐temperature tails and lower temperatures for the start of decomposition. These findings were explained by an increase of available reactive hydrogen at low temperature, probably due to adsorbed H atoms. An admixture of transition metals that are able to dissociate H_2_ molecules lower the starting temperature of metal oxide reduction.^62^ Due to the low equilibrium decomposition pressure of CaCO_3_ at 573 K (0.0015 Pa), it was assumed that, with transition‐metal catalysts, hydrogenation occurred through direct interaction of CaCO_3_ and H atoms to form CH_4_. It is possible that reaction intermediates formed in the CaCO_3_ surface layer, but this assumption has not been validated. Activation energies derived from experiments in an electrobalance were 105 and 111 kJ mol^−1^ for the Ir‐ and Pd‐catalyzed reactions, respectively. The reaction rate increased steadily with increasing H_2_ pressure and remained constant at sufficiently high pressures. Yoshida et al. dedicated this tendency to a transition in reaction kinetics from slightly higher than first order at low pressures to zero order at high pressures. From reaction kinetics, it was suggested that the rate of dissociative adsorption of H_2_ on the metal surface was fast relative to the overall reaction rate.^51^

Jagadeesan et al. extensively studied hydrocarbon formation from various mixed inorganic carbonates and made remarkable conclusions about hydrocarbon selectivity for C_1_–C_3_ chain lengths.^13^, ^52^ In the first study, Jagadeesan et al. examined methane formation from mixed alkaline‐earth‐metal/transition‐metal carbonates at 823 K.^52^ The operating pressure was not stated and the assumption could thus be made that the work was carried out at atmospheric pressure. In a second paper by Jagadeesan et al., however, it was stated that the earlier study was performed at 0.3–0.5 MPa.^13^ The mixed carbonates were prepared by precipitation form aqueous solutions of NaHCO_3_ and had a composition of MeCa(CO_3_)_2_, in which Me was Co, Ni, or Fe in a Me/Ca ratio of 1:1, and Me_1_Me_2_Ca(CO_3_)_3_, in which Me_1_Me_2_ was CoNi, NiFe, or FeCo in a ratio Me_1_/Me_2_/Ca of 1:1:2. Solid decomposition products consisted of nanoparticles of metal dispersed on metal oxide. Hydrogenation of CoCa(CO_3_)_2_ gave CO_2_ and CH_4_ as major gaseous products. As the reaction proceeded, transition‐metal nanoparticles formed on the carbonate surface, which catalyzed the subsequent conversion of CO_2_ to CH_4_. An increasing amount of H_2_ facilitated the formation of transition‐metal nanoparticles through the reduction of metal ions. The carbonates completely decomposed. For CoCa(CO_3_)_2_ at 823 K, the optimal H_2_ flow rate for maximum conversion and CH_4_ selectivity was 8 cm^3^ min^−1^ for 5 h. These conditions applied to all mixed carbonates. Again, the type of transition metal had a leading role on product gas composition. With Co, complete carbonate conversion occurred with a selectivity to CH_4_ of 80 % (20 % CO_2_). All other transition metals and transition‐metal combinations yielded 100 % CH_4_ selectivity at reduced conversion. CO, H_2_O, and coke were not found. Carbonate conversion dropped in the order of NiCa (81 %)>CoNi (77 %)>FeCo (76 %)>NiFe (16 %)>Fe (4 %). Fe exhibited poor conversion. However, in the presence of Fe (Fe, NiFe, FeCo), traces of higher hydrocarbons up to C_3_ formed. The introduction of Pt or K into FeCa(CO_3_)_2_ did not increase the formation of higher hydrocarbons. Because reduced transition‐metal particles appeared to be essential for high CH_4_ selectivity, catalyst nanoparticles were prepared separately by heating freshly prepared transition‐metal carbonates in H_2_. They contained nanoparticles of transition metals, bivalent metal oxide, and CaO and were highly efficient in catalyzing the conversion of CO_2_ to CH_4_. With CoCa(CO_3_)_2_, if mixed in a 50:50 weight ratio with metal–metal oxide nanoparticles, the amount of CH_4_ formed during the first 2 h was four times higher. Studies on the effect of transition‐metal–metal oxide nanoparticles on hydrogenation of mixed carbonates indicated a change in reaction kinetics. In the presence of Co/CaO/CoO catalysts, H_2_ efficiency improved to yield 100 % CH_4_ selectivity for CoCa(CO_3_)_2_, in contrast to 80 % in the absence of the catalyst. The effect of Co on the methanation of carbonates was higher than that of Ni and Fe, and combinations thereof. The catalyst nanoparticles were also capable of decomposing the natural minerals calcite and dolomite. Complete conversion of MgCO_3_ and CaCO_3_ with 100 % CH_4_ selectivity was achieved with Co/CaO/CoO. The type of catalyst significantly influenced the ability to convert CaCO_3_ into CH_4_. Ni and Co, for instance, worked well individually, but were less active upon combination. Fe was not very active itself, but effectively catalyzed CaCO_3_ decomposition in combination with Ni and even more effectively with Co. CaCO_3_ conversion decreased in the order of Co (100 %)>Ni (80 %)>Fe (18 %) for single transition metals and FeCo (89 %)>NiFe (40 %)>CoNi (34 %) for combinations thereof.

In a second study, Jagadeesan et al. directly focused on the formation of C_1_–C_3_ hydrocarbons from CaCO_3_ through iron oxides.^13^ The starting carbonate, denominated FeCaCO, consisted of CaCO_3_ and Fe oxides with Fe/Ca molar ratios, *x*, of 0–5. The carbonate contained Fe in the form of Ca_1−*y*_Fe_*y*_CO_3_ in the calcite structure (for *x*<2). Excess Fe was present as Fe_2_O_3_ and increased with increasing *x*. The effect of reaction temperature (573–873 K) was investigated at ambient pressure and a H_2_ flow rate of 3 cm^3^ min^−1^ in a continuous‐flow packed‐bed reactor. The reaction time was 2 h, after which time no further decomposition of the carbonate occurred. In any case, carbonate conversion was not complete. At 673 K, the carbonate conversion and yield of carbohydrates (23 % at *x=*5) were highest. Further gaseous products were CO_2_ and CO. Iron metal (α‐Fe), iron oxides (Fe_3_O_4_, γ‐Fe_2_O_3_, α‐FeOOH, CaFe_2_O_4_), and carbide (d‐Fe_3_C, χ‐Fe_5_C_2_) particles formed as solid residues supported on Ca‐rich oxides. The level of conversion and yield of hydrocarbons, suggesting higher hydrocarbon selectivity, increased with increasing molar ratios of Fe/Ca. At *x=*5, the yield of the gaseous products CH_4_, C_2_H_4_, C_2_H_6_, C_3_H_8_, CO, and CO_2_ was 5, 7, 4, 4, 6, and 21 %, respectively. In the absence of Fe, mainly CO_2_ formed with traces of CO. Consequently, Fe not only improved carbonate decomposition, but also the subsequent hydrogenation of released CO_2_ to higher hydrocarbons. For all molar ratios, the relative C_2_H_4_ yield was highest among all hydrocarbons. The yield of C_3_H_8_ increased with increasing amounts of Fe, which suggested that higher concentrations of Fe favored C−C coupling, rather than dehydrogenation. The total hydrocarbon yield from Fe‐catalyzed CaCO_3_ hydrogenation was comparable to that in the FT synthesis. A reaction mechanism based on carbonate decomposition to CO_2_, which underwent further reduction to CO and hydrocarbons, was stated. According to Jagadeesan et al., the formation of CO was crucial because CO and H_2_ were adsorbed on the catalyst surface and gave rise to hydrocarbon formation. They speculated that particle size played an important role in the selectivity of the reaction. From FT synthesis, it is known that smaller particles show lower H_2_ chemisorption potential, which favors the formation of olefins instead of C−C coupling.^63^

## 5 Main Benefits

The main benefits of metal carbonate hydrogenation may be summed up as outlined in the following sections.

### 5.1 CO_2_ emission reduction

CO_2_ is the primary greenhouse gas emitted through human activities. In 2015, the global CO_2_ concentration in the atmosphere reached an average value of (399.4±0.1) ppm.^64^ At present, the industrial sector is responsible for approximately one‐third of the total anthropogenic CO_2_^equivalent^ (CO_2_^e^) emissions.^65^ Decarboxylation of metal carbonates under reducing conditions may contribute to a substantial decrease of CO_2_ emissions, especially in high‐emission industrial sectors, such as the iron and steel industry, in which carbonaceous ores are used.^12^ As opposed to conventional decarboxylation processes, CO_2_ is not released into the atmosphere, but reduced to CO, CH_4_, and higher hydrocarbons. The formation of reduced carbon species in the gas atmosphere adds value to the overall process compared with state of the art decarboxylation under oxidizing conditions.

### 5.2 Renewable production of chemicals and fuels

At present, the three major carbon feedstocks are still petroleum, coal, and biomass. Because most of earth's carbon (>99.9 %) exists as carbonates, carbonaceous minerals may provide a potential carbon source for hydrocarbon synthesis in the future. The conversion of carbonaceous inorganic rocks (e.g., calcite, magnesite, dolomite) to organic compounds may help to fulfil future energy requirements and provide a renewable and nearly inexhaustible resource for the production of chemicals.^66^

Because methane naturally occurs in biogas, power generation is still its main purpose. The conversion of biogas to electricity is standard technology. Apart from the production of synthesis gas (syngas) and its utilization, further uses of methane include catalytic and noncatalytic oxidative coupling (OCM) to C_2+_ hydrocarbons, direct oxidation to methanol or formaldehyde, oxidative methylation of hydrocarbons by methane, and oxidative carboxylation of methane by CO to acetic acid.^67^ The generation of H_2_ from CH_4_ is industrially accomplished through steam methane reforming, but the conversion can also be achieved by pyrolysis.^68^, ^69^, ^70^

From syngas, which is the feed material for a FT process, a synthetic crude oil (syncrude) is obtained. The syncrude consists of a multiphase mixture of hydrocarbons, oxygenates, and water. Refining of the syncrude yields products that are traditionally produced from conventional crude oil, such as transportation fuels and chemicals.^21^, ^71^

### 5.3 Hydrogen storage

Hydrogenation of metal carbonates can also be seen as a means of chemical hydrogen storage in the form of CH_4_ 72 or fuels produced from syngas.^73^, ^74^ This is similar to the power‐to‐methane (PtM) concept that converts electrical into chemical energy by using captured CO_2_ and H_2_ from water electrolysis.^75^, ^76^ The main advantage of these alternative hydrogen‐storage technologies is the availability of storage and distribution systems prepared for natural gas and liquid hydrocarbon fuels. In regions where a natural gas infrastructure exists, both concepts provide a promising option to absorb and exploit surplus renewable energy.

### 5.4 Catalyst preparation

Tailor‐made solid products, regarding composition and morphology, may be achieved through adjusting the process conditions, especially the gas atmosphere. Morphology plays a crucial role if the solid products represent catalytically active materials, both catalytically active material and support material.^49^ A hydrogen atmosphere opens up a new pathway for transition‐metal carbonate transformation into finely dispersed, active catalysts for in situ or ex situ use.^10^ Although initial morphological studies seem promising, the catalytic activity of reductively calcined material has not yet been tested. To the best of our knowledge, only reductively calcined MgO was studied for its catalytic properties for CO_2_ conversion with H_2_, revealing reverse water‐gas shift activity.^53^ Long‐term stability was not considered.

### 5.5 Chemical solar energy storage

Experiments with energetic light, for instance Vis or UV radiation, indicate that irradiation affects the course of metal carbonate decarboxylation.^10^ The mechanistic course could depend on the wavelength of the radiation source, giving access to new pathways for the application of solar energy in solar furnaces. In principle, solar energy can be stored and transformed by means of cyclic inorganic processes. Primary electron spectroscopy for chemical analysis (ESCA) experiments revealed the difference between thermal hydrogenation and hydrogenation induced by irradiation.^22^ The results confirmed an effect of the type of energy (UV/Vis radiation) on the mechanism and kinetics of decomposition, but no precise conclusion was feasible and no further reports were made.^77^

## 6 Potential Fields of Application

Two fields of application were identified and analyzed regarding potential, limitations, and domains requiring further research. Both elucidate the need for further studies in industrial‐sized reactors, allowing appropriate process conditions to be stated for industrial application.

### 6.1 CCU based on mineral carbonation

Mineral carbonation is a novel, widely investigated concept for CO_2_ capture and storage based on weathering of limestone in nature.^9^, ^78^, ^79^, ^80^, ^81^, ^82^, ^83^ In mineral carbonation, high concentrations of captured CO_2_ from an industrial or power‐sector source react with metal oxide [MeO, mainly CaO or MgO; Eq. (28)] or metal hydroxide bearing materials to form the corresponding insoluble carbonate.

(28) $$\mathrm{MeO}+\mathrm{CO}{}_{2}\to \mathrm{MeCO}{}_{3}+\mathrm{heat}$$

Due to the lower energy state of inorganic carbonates relative to CO_2_, the reaction is exothermic. Therefore, in theory, the process does not require any energy input, but produces heat. Unfortunately, extensive preparation of the solid reactants (including mining, transportation, grinding, and activation, if necessary); the use, recycling, and loss of additives and catalysts; and disposal of carbonates and byproducts render the overall process energy intensive and require external high‐grade energy sources.^84^ Due to its thermodynamics, carbonate formation is favored at low temperatures. High temperatures favor the reverse reaction, namely, decarbonation, which is generally referred to as calcination.

Appropriate carbonaceous feedstock sources include abundant silicate rocks that involve laborious mining and alkaline industrial residues that are readily available, but only on a small scale (e.g., slag from steel production or fly ash).^85^ Pure calcium and magnesium oxides and hydroxides provide the ideal source material because they are more readily carbonated than that of the corresponding silicates. However, due to their high reactivity, they are scarce in nature.^78^, ^84^

There are several single (direct) or multistep (indirect) dry or wet process routes. In aqueous environment, carbonation is faster, but, due to higher dilution and lower reaction temperatures, the heat of reaction is difficult to retrieve. The dry process is a simpler approach that brings gaseous CO_2_ into contact with particulate metal oxide bearing materials. Easy recovery of the heat of reaction is beneficial for this method, but the bottleneck is the slow rate of reaction at suitable temperature levels. It is only feasible at elevated pressures for refined, rare materials, such as the oxides and hydroxides of calcium and magnesium.^9^, ^84^

The generated carbonates (CaCO_3_, MgCO_3_), if not disposed of, are used for mine reclamation or in construction.

It is expected that alkaline‐earth‐metal carbonates will give access to reversible thermal decarbonation/recarbonation cycles if decarbonation is carried out under a reducing hydrogen atmosphere.^55^ In H_2_, CO_2_ is not released into the flue gas, but further reduced to CO.^86^ Reductively calcined MgO, CaO, SrO, and BaO were found to be constituted of solid conglomerates of microcrystalline domains featuring pronounced reactivity towards recarbonation; a fact that renders them promising as potential CO_2_‐trapping systems.^48^ Repeated carbonation/recarbonation cycles omit excessive measures for reactant preparation, makeup of additives and catalysts, and product disposal. Consequently, the net energy input required is potentially lower. Once prepared, refined, small particles of metal oxides can be repeatedly used; this poses a pronounced advantage for the reaction of dry CO_2_ gas with solid oxides not only as far as labor input is concerned, but also for the rate of reaction. It is well known that small particle sizes facilitate high reaction rates.^78^ In conventional mineral carbonation of CO_2_, most of the energy required is needed for grinding of the feedstock to particles of 100 μm. During carbonation, the formation of silica and carbonate layers on the mineral surface hinders the reaction and limits conversion. Carbonate layers are barely prevented, but silica layers do not form because pure metal oxides are applied. Conventional CO_2_ mineralization is criticized for its tremendous environmental impact associated with large‐scale mining directly leading to land clearing and product disposal; an issue that does not need any consideration if the metal oxides are repeatedly used.

**Figure 3:**
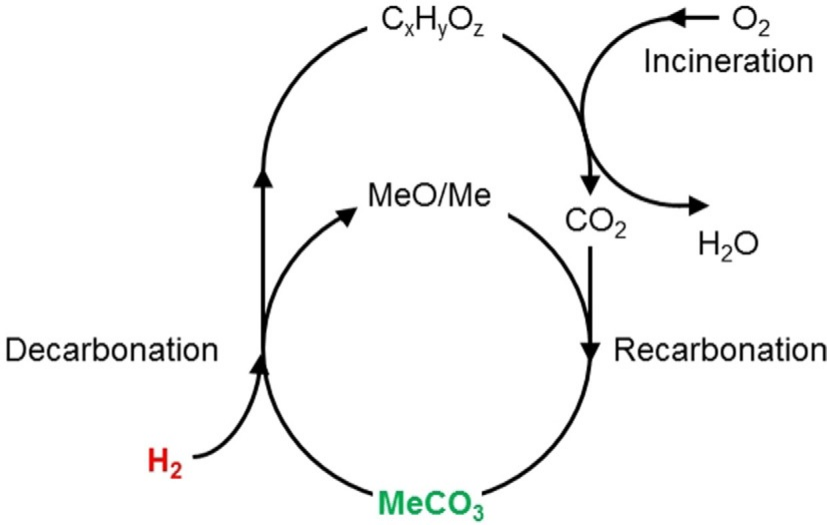
Concept of a closed CO_2_ circuit based on decarboxylation (decarbonation) of metal carbonates MeCO_3_ through hydrogenation followed by recarbonation (adapted from Ref. 55).

Through transition‐metal doping of the solid reactant, a composite system may be generated, in which CO_2_ is trapped on metal oxides (carbonation/recarbonation step) and subsequently transformed into higher organic species through hydrogenation of the metal carbonate (decarbonation step; Figure 3).^55^

The technology for mineral carbonization is still immature. The concept of a closed CO_2_ circuit based on decarbonation through hydrogenation followed by recarbonation is a promising concept. Nevertheless, it is merely based on primary laboratory‐scale experiments and further research is required for a feasible economic analysis.

For a new 600 MW_e_ coal‐fueled power plant with an annual CO_2_ emission of 4 Mt year^−1^, a total energy requirement of 580 kWh for CO_2_ capture and carbonation has been estimated.^9^ Because most of the energy is needed for grinding of the feedstock material (280 kW h) and this step is omitted for repeated decarbonation/recarbonation cycles, power plant efficiencies may increase from 23.6 to 33 % and CO_2_ avoidance rates from 72.5 to 82 % for the concept of a closed CO_2_ circuit.^9^ In the literature, reported cost estimations of various carbonation routes differ significantly. At present, direct aqueous technologies seem to be the most realistic ones with costs ranging from € 60 to 100 t^−1^ CO_2_ fixed.^87^ Additional CO_2_ emissions associated with the energy required for the carbonation process will boost the costs to € 80 to 130 t^−1^ CO_2_ fixed. Further taking into account the costs for capturing CO_2_ from a power plant yields total costs of € 150 t^−1^ CO_2_ avoided for a full CCS system with mineral carbonation.^9^

At this point, composite systems derived from transition‐metal doping that may not only trap CO_2_, but also transform it into higher organic species are not considered. Further energy, and consequently, cost savings are expected due to increasing reaction rates of carbonation based on the use of refined metal oxide reactants with small particle sizes. Metal doping also allows for lower reaction temperatures for the carbonation step and decreasing activation energies. Furthermore, dry carbonation will allow for easy accessibility of the heat of reaction of carbonation. Currently, cost estimation is not feasible because the type of catalyst, hydrocarbon species generated, and hydrocarbon selectivity still need to be established. Initial studies highlight its potential and elucidate the need for further research into process conditions and the long‐term stability of the system, which are crucial factors for its economic viability.

### 6.2 Direct reduction of mineral iron carbonate

Austria and China have major siderite reserves for iron and steel production. Siderite beneficiation is challenging because of the low iron content of the ore compared with magnetite and hematite ores. The industrial practice is to blend siderite with other high‐grade ores in the sinter plant. During the sintering process, siderite is converted into hematite through roasting in air. The sinter product is fed to the blast furnace (BF), in which it is preferably reduced with coke via CO, producing at least 1.5 mol CO_2_ per mole of iron due to the stoichiometry of reaction. Consequently, at least 2.5 mol CO_2_ are emitted during the production of 1 mol iron from iron carbonate.

**Figure 4:**
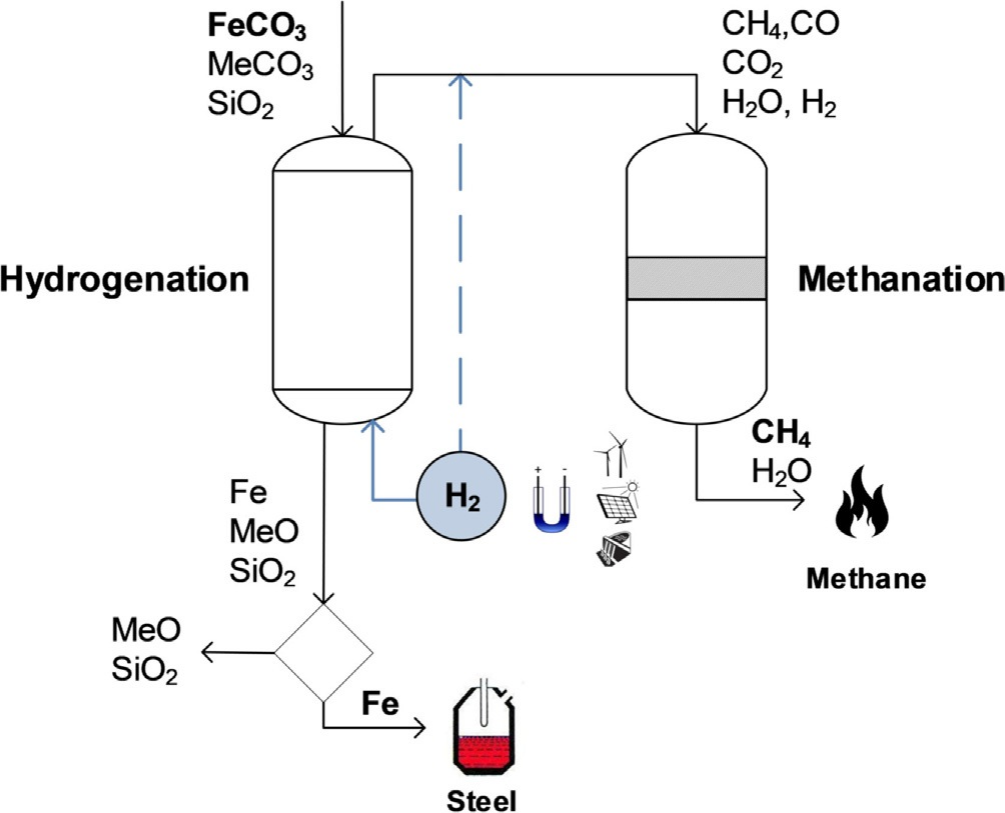
Process concept for direct iron production from the mineral siderite followed by off‐gas valorization by catalytic CO_2_ hydrogenation, for example, methanation.

Direct hydrogen reduction of the mineral iron carbonate represents a novel process concept for sustainable pig iron production. It is a high‐potential approach for significant energy savings and CO_2_ emission reduction, especially if coupled with catalytic CO_2_ hydrogenation (e.g., methanation) to further convert inevitably released CO_2_ (Figure 4).^12^ Due to the debate about a sustainable energy supply, research into methanation has increased tremendously in recent years and is readily available.^88^, ^89^, ^90^, ^91^, ^92^, ^93^, ^94^, ^95^

#### 6.2.1 Proof of concept

TG studies revealed that mineral iron carbonate was directly reduced to elemental iron under a hydrogen atmosphere. Iron carbonate reduction was represented by a distinct mass loss below 723 K, which was followed by a small relative mass loss spanning over a broad temperature range (723–923 K) allocated to the concomitant decomposition of manganese, magnesium, and calcium carbonate to the respective oxides.^12^

**Figure 5:**
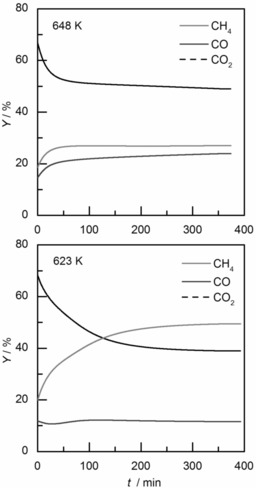
Dry product gas composition for the direct hydrogen reduction of the mineral siderite at 623 and 648 K; 60 g siderite, size fraction: 0.5–1 mm, H_2_/N_2_=9:1, 867 cm^3^ min^−1^, ambient pressure.

In the ideal case of complete carbonate conversion, elemental iron is formed together with CO_2_, CO, CH_4_, and potentially even higher hydrocarbons (C_*x*_H_*y*_). Baldauf‐Sommerbauer et al. investigated the effect of temperature (Figure 5) and pressure on the composition of the product gas that consisted of CO_2_, CO, and CH_4_.^96^ Elevated pressure and low temperature increased the yield of CH_4_. CO formation was preferred at low pressure and higher temperatures.

CO_2_ emission savings of at least 60 % are possible because, at most, 1 mol CO_2_ is released per mole of elemental iron. If hematite is reduced with hydrogen, 1.5 mol hydrogen is required per mole of elemental iron. Consequently, up to 33 % less reducing agent is needed if direct siderite reduction is applied, due to circumventing the hematite route. Direct siderite reduction can be run at relatively low temperatures (673–773 K) compared with other metallurgical iron carbonate beneficiation processes, such as the Midrex^©^ process for direct iron oxide reduction with natural gas (1053–1073 K)^97^ or the classical BF process (1773 K).^98^

#### 6.2.2 Case studies

A comparison of four different case studies with the state‐of‐the art BF process highlights the potential of the concept of direct hydrogen reduction of the mineral iron carbonate.

As feed material, the mineral siderite with a characteristic composition of a concentrated siderite sample from the Styrian Erzberg in Austria (Table 2) was chosen. Iron was assumed to occur in the form of FeCO_3_ (79 wt %) and CaFe(CO_3_)_2_ (5 wt %). Minor components were MgCO_3_, MnCO_3_, SiO_2_, and Al_2_O_3_.

**Table 2:** Mean composition of the mineral siderite from the Styrian Erzberg in Austria.

Component	Mass fraction
FeCO_3_	0.79
CaFe(CO_3_)_2_	0.05
MgCO_3_	0.07
MnCO_3_	0.05
SiO_2_	0.03
Al_2_O_3_	0.01

The classical BF process serves as benchmark. It consists of a sintering step under oxidizing conditions [Eqs. (29), (30), (31), (32), (33), 1373 K) in which FeCO_3_ is transformed into Fe_2_O_3_ for reduction in the BF [Eq. (34), 1773 K].

(29) $$\mathrm{FeCO}{}_{3}\to \mathrm{FeO}+\mathrm{CO}{}_{2}$$

(30) $$2\;\mathrm{FeO}+0.5\;O{}_{2}\to \mathrm{Fe}{}_{2}O{}_{3}$$

(31) $$\mathrm{MgCO}{}_{3}\to \mathrm{MgO}+\mathrm{CO}{}_{2}$$

(32) $$\mathrm{MnCO}{}_{3}\to \mathrm{MnO}+\mathrm{CO}{}_{2}$$

(33) $$\mathrm{CaFe}\left(\mathrm{CO}{}_{3}\right){}_{2}\to \mathrm{CaO}+\mathrm{FeO}+2\;\mathrm{CO}{}_{2}$$

(34) $$\mathrm{Fe}{}_{2}O{}_{3}+3\;C\to 2\;\mathrm{Fe}+3\;\mathrm{CO}$$

During direct hydrogen reduction to elemental iron, several reactions are conceivable for FeCO_3_ [Eqs. (35), (36), (37)] and CaFe(CO_3_)_2_ [Eqs. (38), (39)], depending on the product gas. The reaction temperature was set to 773 K.

(35) $$\mathrm{FeCO}{}_{3}+2\;H{}_{2}\to \mathrm{Fe}+\mathrm{CO}+2\;H{}_{2}O$$

(36) $$\mathrm{FeCO}{}_{3}+5\;H{}_{2}\to \mathrm{Fe}+\mathrm{CH}{}_{4}+3\;H{}_{2}O$$

(37) $$\mathrm{FeCO}{}_{3}+H{}_{2}\to \mathrm{Fe}+\mathrm{CO}{}_{2}+H{}_{2}O$$

(38) $$\mathrm{CaFe}\left(\mathrm{CO}{}_{3}\right){}_{2}+2\;H{}_{2}\to \mathrm{CaCO}{}_{3}+\mathrm{Fe}+\mathrm{CO}+2\;H{}_{2}O$$

(39) $$\mathrm{CaFe}\left(\mathrm{CO}{}_{3}\right){}_{2}+5\;H{}_{2}\to \mathrm{CaCO}{}_{3}+\mathrm{Fe}+\mathrm{CH}{}_{4}+3\;H{}_{2}O$$

To calculate CO_2_ emissions, direct emissions of CO_2_ and CO are summed due to CO oxidation to CO_2_ [Eq. (40)]. Oxidation of CO contributes to CO_2_ emission, but reduces the total energy demand. A reduction in energy demand because of CH_4_ was also considered. Hydrogen supply was assumed to be accomplished by water electrolysis (4.8 kWh Nm^−3^).^24^, ^25^

(40) $$\mathrm{CO}+0.5\;O{}_{2}\to \mathrm{CO}{}_{2}$$

Four case studies, Red1, Red2, Red3, and Red4, represent the extreme cases (Red1 and Red2) and mixed cases (Red3 and Red4). In Red1, full carbon conversion to CO was assumed [Eqs. (35) and (38)]. CH_4_ formation was postulated for Red2 [Eqs. (36) and (39)]. For Red3, 50 % CO and 50 % CH_4_ were assumed. Red4 reproduces the experimental product composition depicted in Figure 5 (49 % CO_2_, 27 % CH_4_, 24 % CO).

Total CO_2_ emissions and the total energy demand for all four cases are compared with the benchmark BF process in Table 3. The results quantify the capability of CO_2_ emission reduction. The classical BF process releases 2212 kg CO_2_ t^−1^ pig iron. 100 % CO formation (Red1) saves 64 % of the CO_2_ emitted in the benchmark process. No CO_2_ is released if full conversion to CH_4_ is hypothesized (Red2). This scenario exhibits the highest energy demand of 5267 kW h (111 % compared to the BF process) and is not aspired to from an economic point of view. Scenarios with CO and CH_4_ (Red3 and Red4) show excellent CO_2_ emission reduction (82 and 74 %) and decreased energy demand (11 and 28 %, although a hydrogen supply from water electrolysis was chosen), which underlines the high potential of the proposed concept.

**Table 3:** Total CO_2_ emission and total energy demand for the case studies Red1 (100 % CO formation), Red2 (100 % CH_4_ formation), Red3 (50 % CO and 50 % CH_4_), and Red4 (49 % CO_2_, 27 % CH_4_, 24 % CO) compared with the benchmark BF process.

Case study	CO_2_ emission	Energy demand	
	[kg CO_2_ t^−1^ Fe]	[GJ t^−1^ Fe]	[kWh]
BF	2212	17.1	4755
Red1	788.5	11.4	3156
Red2	0	19.0	5267
Red3	394.3	15.2	4211
Red4	569.9	12.2	3401

Direct iron carbonate reduction is a high‐potential candidate to open up a new route for environmentally benign pig iron production. The findings are based on TG experiments with siderite^12^ and tests in a tubular reactor setup;^96^ thus direct conclusions for application in large‐scale reactors and optimized process conditions (e.g., particle size, temperature) cannot be drawn yet. Iron separation from the unconverted siderite matrix and gangue through magnetic separation was suggested in the literature, but still needs verification. Nevertheless, the presented case studies highlight the potential of reductive calcination of siderite and the need for ongoing research in this field.

## 7 Summary and Outlook

Various aspects render metal carbonate hydrogenation a powerful means for direct and indirect CO_2_ emission reduction, CO_2_ utilization, and metal carbonate exploitation.

Under a hydrogen atmosphere, the decarboxylation temperature is significantly lower than that of the respective reaction under inert conditions. Doping with transition metals further lowers the temperature level. The combination of decarboxylation and CO_2_ reduction with the renewable energy carrier hydrogen transforms the conventional endothermic process into an overall exothermic process, which allows for significant energy savings.

In reductive metal carbonate decarboxylation, CO_2_ is not (or only partially) released, but reduced to CO, CH_4_, and higher hydrocarbons. The composition of the gaseous reaction product strongly depends on the gas atmosphere (pure or dilute hydrogen); the presence of transition‐metal species acting as in situ catalysts; and the reaction temperature, pressure, and residence time. Apart from metal oxides in various oxidation states, elemental metals are obtained as solid reaction products from transition‐metal carbonates. Tailormade products, in terms of composition and morphology, would give access to novel production routes for catalysts.

Until now, preliminary studies focusing on feasibility and chemism have mainly been made with metal carbonates in small‐scale apparatus, lacking transferability to industrial scale. Additionally, disagreement exists concerning the reaction mechanisms. Whereas some researchers propose the direct reaction of hydrogen with fixed CO_2_ in the carbonate, others assume that hydrogen reacts with released CO_2_. Degradation studies in hydrogen and nitrogen revealed differences in morphology that indicate the direct reaction of H_2_ with CO_2_; however, several aspects require closer examination, especially when it comes to optimized process conditions for industrial applications. Once clarified, metal carbonate hydrogenation could provide a quantum leap in high‐emission industrial sectors, such as the iron and steel industry, if a renewable hydrogen supply is accomplished. Further potential fields of applications include the renewable production of chemicals and catalyst preparation.

## Conflict of interest

*The authors declare no conflict of interest*.

## Biographical Information

Susanne Lux studied technical chemistry at the Graz University of Technology in Austria. In 2009, she obtained her Ph.D. in chemical engineering under the supervision of Prof. Matthäus Siebenhofer. In 2012, she accepted a position as Assistant Professor at the Institute of Chemical Engineeering and Environmental Technology at Graz University of Technology. Her research group focuses on process intensification, with the main focus on reactive separation technologies and heterogeneous catalytic systems to address environmental challenges.

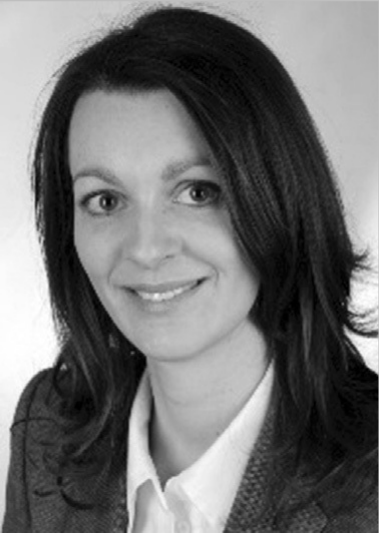

## Biographical Information

Georg Baldauf‐Sommerbauer studied chemistry (B.Sc.) and chemical engineering (M.Sc./Dipl.‐Ing.) at the University of Graz and Graz University of Technology in Austria. In 2017, he successfully defended his Ph.D. thesis (supervised by M. Siebenhofer and S. Lux) on the reductive calcination of mineral carbonates. His research interest focuses on fundamental kinetic studies, as well as on applied studies towards a possible industrial implementation of renewable technologies.

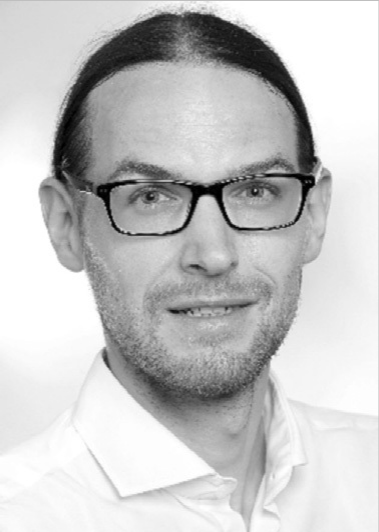

## Biographical Information

Matthäus Siebenhofer studied chemical engineering and received his doctoral degree in 1983 from the Graz University of Technology in Austria. Currently, he is Professor for Chemical Reaction Engineering and Head of the Institute of Chemical Engineering and Environmental Technology at Graz University of Technology.

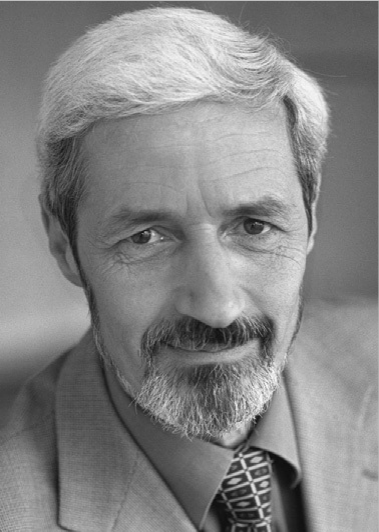
